# Kombination von simulationsbasiertem Lernen und Online-Learning in der Augenheilkunde: Effizienz von Simulation in Kombination mit eigenständigem Online-Learning im Rahmen von EyesiNet in der studentischen Ausbildung

**DOI:** 10.1007/s00347-020-01313-0

**Published:** 2021-01-15

**Authors:** Svenja Deuchler, Christina Sebode, Hanns Ackermann, Ingo Schmack, Pankaj Singh, Thomas Kohnen, Frank Koch

**Affiliations:** 1 Augenklinik, Klinikum der Johann Wolfgang Goethe-Universität Frankfurt, Theodor-Stern-Kai 7, 60590 Frankfurt am Main, Deutschland; 2 Institut für Biostatistik und Mathematische Modellierung, Johann Wolfgang Goethe-Universität, Frankfurt am Main, Deutschland

**Keywords:** EyesiNet, Student, Augenerkrankungen, Netzhaut, Lehre, EyesiNet, Student, Eye diseases, Retina, Education

## Abstract

**Hintergrund:**

Die Ophthalmoskopie ist Bestandteil des medizinischen Curriculums, jedoch das Vermitteln der Inhalte oft unbefriedigend, da ein systematisches Lernen von Pathologien und deren Behandlungen dadurch erschwert ist, dass oftmals das passende Patientengut nicht zur Verfügung steht und dadurch gesunde Studenten einander untersuchen müssen. Aus diesem Grund haben wir eine Online-Plattform entwickelt, die in Kombination mit simulationsgestütztem Training sowohl das eigenständige als auch das angeleitete Lernen von Untersuchungsmethoden und Pathologien ermöglicht.

**Ziel der Arbeit:**

Ziel der vorliegenden Arbeit war, ein Format für die Verbesserung der Lehre der direkten und indirekten Ophthalmoskopie im Studierendenunterricht zu evaluieren. Dabei wurden praktische Übungen an Virtual-Reality-basierten Simulatoren mit neu entwickelten und an den Lehrkatalog angepassten theoretischen Inhalten in der Online-Plattform EyesiNet verschränkt.

**Material und Methoden:**

Die Studierenden bearbeiteten am ersten sowie am letzten Praktikumstag zufällig ausgewählte Fälle, die ihnen von den Eyesi Direct- und Eyesi Indirect-Simulatoren präsentiert wurden. Zwischen diesen beiden Einheiten konnten sie sich auf freiwilliger Basis mit den theoretischen Grundlagen typischer ophthalmologischer Krankheitsbilder im EyesiNet beschäftigen.

**Ergebnisse:**

*Eyesi Direct*: Die Bewertung des Simulators ergab am ersten Praktikumstag für beide Gruppen keinen signifikant unterschiedlichen Wissensstand (*p* = 0,29). In der Gruppe OHNE Training (*n* = 54) ergab sich am letzten Praktikumstag mit *p* = 0,02 eine signifikante Verbesserung dieser Bewertung, jedoch mit einer geringen Effektgröße von 0,1. In der Gruppe MIT Training (*n* = 32) konnte mit *p* = 0,0004 eine hoch signifikante Verbesserung mit einer Effektgröße von 0,3 nach Rosenthal festgestellt werden. *Eyesi Indirect*: Die simulatorgestützte Bewertung ergab am ersten Praktikumstag keinen signifikanten Unterschied im Wissensstand der beiden Gruppen (*p* = 0,1). Nach dem Training schnitten zwar beide Gruppen etwas besser ab, jedoch ohne signifikanten Unterschied (OHNE Training *p* = 0,41/MIT Training *p* = 0,17).

**Diskussion:**

Die Online-Plattform EyesiNet unterstützt beim Erlernen der wichtigsten Erkrankungsbilder. Lerninhalte werden reproduzierbar und auf für alle Lernenden standardisierte Weise zur Verfügung gestellt. Die Fertigkeiten der direkten Ophthalmoskopie sind hierbei deutlich schneller als die der indirekten Ophthalmoskopie zu erlernen.

## Abstract (en)

**Background:**

Ophthalmoscopy is part of the medical curriculum but the teaching of medical contents is often unsatisfactory. No systematic learning of pathologies and their treatment options is possible because appropriate patient populations are often unavailable; instead of that healthy students examine each other. For this reason, we have developed a project to improve teaching at the medical faculty of the JWG University in Frankfurt/Main, which offers the opportunity for training in ophthalmoscopy using a newly developed online platform (EyesiNet) in addition to simulator training. Defined learning contents are reproducible and made equally available to everyone.

**Aim:**

The aim of this study was to evaluate a format for improving the teaching of direct and indirect ophthalmoscopy for students. Practical exercises on virtual reality-based simulators were intertwined with newly developed theoretical content adapted to the teaching catalog in the online platform EyesiNet.

**Material and methods:**

On the first and last days of the internship, the students worked on cases in Eyesi Direct and Eyesi Indirect. In the meantime, they were able to deal with the theoretical basics of these ophthalmological clinical pictures in the web-based EyesiNet on a voluntary basis.

**Results:**

*Eyesi Direct*: with *p* = 0.29, both groups had the same state of knowledge and did not have significantly different results in case processing on the first day of the internship. In the group without training (*n* = 54), a significant improvement in simulator training was observed on the last day of the practical training with *p* = 0.02 but with a small effect size of 0.1. Among the trainees (*n* = 32) a highly significant improvement with an effect size of 0.3 was observed with *p* = 0.0004. *Eyesi Indirect*: both groups had no significant difference in the stage of knowledge in the Eyesi Indirect at the beginning of the training period (*p* = 0.10). After training both groups performed slightly better but without significance (without training *p* = 0.41/with training *p* = 0.17).

**Conclusion:**

The online platform EyesiNet supports the learning of the most important disease patterns. Learning contents are made available in a reproducible and standardized way for all students. The skills of direct ophthalmoscopy can be learned much faster than those of indirect ophthalmoscopy.

Direkte und indirekte Ophthalmoskopie sind fester Bestandteil des medizinischen Curriculums. Im Rahmen eines Projekts zur Verbesserung der Lehre am Fachbereich Medizin der Johann Wolfgang Goethe-Universität Frankfurt am Main haben wir ein Lernformat entwickelt, das praktische Übungen an Simulatoren mit theoretischer Wissensvermittlung innerhalb eines Online-Zugangs („EyesiNet“) kombiniert und dabei die Inhalte des Lehrkatalogs des Medizinstudiums berücksichtigt.

## Hintergrund und Fragestellung

Wie wichtig es für alle Studierende ist, Basisfertigkeiten in der Ophthalmologie zu erlernen, konnte eine Umfrage von 93 Studierenden zeigen [1]; 53 % dieser Gruppe sind später neben der Ophthalmologie in Fachbereichen wie Innere Medizin, Pädiatrie, Gynäkologie, Allgemeinmedizin, Neurologie oder Notfallmedizin tätig, in denen ein Screening des Auges eine essenzielle Fertigkeit darstellt. Die Ausbildung mithilfe von echtem Instrumentarium wird jedoch seit Jahren dadurch erschwert, dass zum Zeitpunkt des Praktikums nicht genügend Patienten mit verschiedenen Erkrankungsbildern zur Verfügung stehen und darüber hinaus Zeitdruck in den Ambulanzen besteht. Die typische Alternative – die Auszubildenden untersuchen sich gegenseitig – ist meist nicht kompatibel mit deren Tagesablauf (Pupillenerweiterung, Lese- und Fahruntüchtigkeit), zudem wird hierdurch systematisches Lernen von Pathologien und deren Behandlungsoptionen [2] erschwert. Für eine systematischere und effektivere Lehre wurden bereits verschiedene Anstrengungen unternommen. So wurde neben dem klinischen Training von Lippa et al. [1] der Fundoskopiesimulator CLEO (Clinical Learning Experience in Ophthalmoscopy) verwendet. CLEO ist ein anatomisch korrekter Simulator, bei dem ein Schaufensterpuppenkopf mit einem erweiterten und einem nicht erweiterten Auge verwendet wird. Die Netzhäute dieser beiden Modellaugen werden durch einen Diabetrachter mit dem Dia einer Fundusfotografie (35-mm-Weitwinkeloptik [60°]) mit bekannter Pathologie simuliert [1].

Kelly et al. [3] beschreiben ein Styropormodell, bei dem fotografische Aufnahmen echter Retinae auf dem Innenboden eines weißen Polyethylenzylinders (ähnlich eines Kleinbildfilmdöschens) angebracht sind. Eine Linse, die in die Öffnung des Polyethylenzylinders eingesetzt ist, reproduziert die optischen Abbildungsverhältnisse des echten Auges. Das Training verschiedener Erkrankungsbilder mithilfe dieser Anordnung führt zu einer signifikanten Leistungsverbesserung.

Die Möglichkeit, typische Krankheitsbilder anhand von Fundusfotografien zu erlernen, wird mit den Simulatoren Eyesi Direct und Eyesi Indirect mit Virtual-Reality-Techniken weitergeführt und mithilfe der von uns entwickelten Online-Plattform EyesiNet um klassische text- und bildbasierte Lehrinhalte ergänzt. In dieser Kombination werden definierte Lerninhalte reproduzierbar und für alle gleichermaßen zur Verfügung gestellt, sodass – unabhängig von personellen Schwankungen – allen Auszubildenden die gleichen Möglichkeiten geboten werden. Der durch das standardisierte Training erzielte individuelle Lernerfolg wird mess- und vergleichbar. Ein sehr nützlicher Nebeneffekt des computergestützten Trainings ist, dass die Studierenden im Bedarfsfall ihren Lernprozess unabhängig von Lehrveranstaltungsorten und festgelegten Zeiten eigenverantwortlich weiterführen können.

Der angehende Mediziner lernt, typische Krankheitsbilder auf Abruf kennenzulernen und diese ggf. später in Diensten und in Stresssituationen – unabhängig von der Fachdisziplin – wiederzuerkennen, richtig einzuschätzen und die Behandlung korrekt einzuleiten. Dies kann je nach Erkrankungsbild ausschlaggebend für den Erhalt der Sehkraft werden.

## Eyesi Direct und Indirect-Simulator

**Figure 1:**
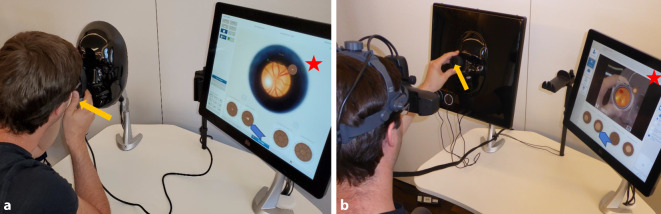

Die von uns verwendeten Simulatoren Eyesi Indirect und Eyesi Direct (Firma VRmagic, Mannheim, Deutschland, Softwareversion 1.8) bestehen aus der Nachbildung des jeweiligen Instruments (direkte bzw. indirekte Ophthalmoskopie mit binokularer Hutophthalmoskopie), einem Patientenmodell, einem Touchscreen-Monitor sowie einem PC (Abb. 1).

Im Okular des simulierten direkten Ophthalmoskops (Eyesi Direct) sieht der Untersuchende eine rein virtuelle Darstellung der beobachteten Strukturen, insbesondere des Augenhintergrunds.

Im Unterschied dazu erfolgt bei Eyesi Indirect die Darstellung mittels virtueller und erweiterter Realität („virtual and augmented reality“), d. h. der Untersuchende sieht das reale Bild seiner Umgebung über ein Videosignal auf Displays im Hutophthalmoskop, allerdings wird anstelle der physisch vorhandenen Patientenmaske ein virtueller Patient in das Bild gemischt. Auf diese Weise kann der Untersuchende seine eigene Hand unter Ophthalmoskopsicht weiterhin kontrollieren und – für diese Untersuchungsmethode sehr wichtig – sich unter Sicht am Kopf des Patienten abstützen, um eine stabile Position für die Untersuchungslupe zu finden. In dieser Mischung aus echter und virtueller Situation entsteht eine realistische und dynamische 3‑D-Lernumgebung.

Bei beiden Simulatoren werden die virtuellen Patienten auf täuschend echte Weise dargestellt. Passend zum dargestellten Erkrankungsbild können sie unterschiedlichen Alters und ethnischer Herkunft sein. Durch die korrekte Bedienung des Ophthalmoskops (Einstellung von Licht und Refraktionsausgleich, Abstand vom Patientenauge, Rotreflex, ggf. Positionierung und Orientierung der Ophthalmoskopierlupe) ist es möglich, ein realistisches Bild der Retina zu sehen [4, 5]. Die von der Software angezeigten Fallbeschreibungen wurden im Rahmen des Projekts an die Bedürfnisse der Studierenden angepasst und mit den theoretischen EyesiNet-Inhalten abgeglichen: Neben einer kurzen Darstellung der Anamnese des virtuellen Patienten, seines Sehvermögens und Augeninnendrucks sind es v. a. die Multiple-Choice-Fragen für Befundung und Diagnose, die Bezug auf die Krankheitsbilder nehmen, die im webbasierten Theorieteil beschrieben werden (EyesiNet, s. unten).

## EyesiNet

Der Hersteller der Simulatoren stellt bereits ein webbasierte Schulungsportal zur Verfügung (VRmNet, Version 8.0), bei dem die Teilnehmer nach dem Einloggen von einem beliebigen Computer oder Mobilgerät aus Zugang zu einem Orientierungskurs für die Simulatoren haben, ihre Trainingsdaten einsehen können und eine Bibliothek mit ihren am Eyesi Direct und Indirect gefundenen anatomischen und pathologischen Befunden im Laufe des Trainings anlegen können.

**Figure 2:**
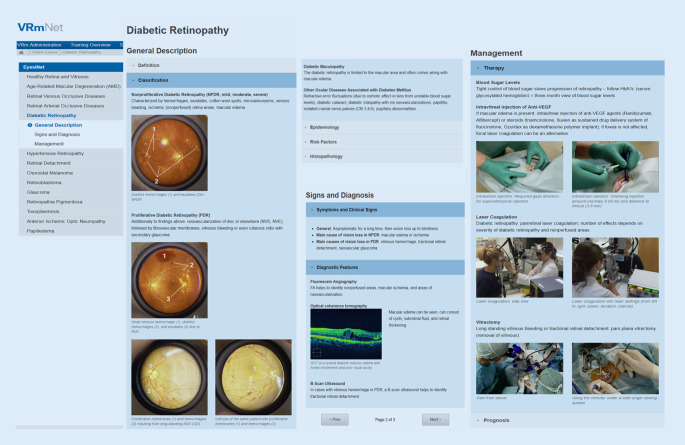

Die medizinischen Inhalte dieser Online-Plattform wurden von uns im Rahmen des Lehrprojektes weiterentwickelt und so strukturiert, dass sie die Bedürfnisse der Studierenden und den Lernzielkatalog widerspiegeln (z. B. hypertensive Retinopathie, diabetische Retinopathie, Aderhauttumore) und auch für die Spezialisierung in anderen Fachgebieten relevant sind (Innere Medizin, Gynäkologie, Pädiatrie, Neurologie u. a.). Hierzu wurden in der von uns angepassten Plattform („EyesiNet“) 14 Fälle und deren Pathologien im Karteikartenformat nach Definition, Klassifikation, Epidemiologie, Risikofaktoren, Histopathologie, Symptomen und klinischen Zeichen, Diagnostik, Therapie und Prognose gegliedert und die jeweiligen Unterpunkte mit Bildern versehen (Abb. 2).

Das Besondere ist, dass die dargestellten Pathologien in EyesiNet anhand von Screenshots aus den Simulatoren erklärt werden, sodass während der praktischen Übungen an simulierten Patienten die Studierenden diejenigen Befunde wiederfinden, die sie sich zuvor im Netz angeschaut haben. Auf diese Weise sind der Wiedererkennungseffekt und die damit einhergehende Lernmotivation deutlich höher. Jeder Student erhält seinen eigenen EyesiNet- und Simulatorzugang. Mithilfe der Online-Inhalte kann das simulatorgestützte Training zu Hause per PC oder Smartphone vor- und nachbereitet sowie der individuelle Trainingsfortschritt überprüft werden.

## Studiendesign und Untersuchungsmethoden

Nach Erstellen der notwendigen Lerninhalte und deren Integration in die Online-Plattform wurde eine prospektive Studie durchgeführt, genehmigt durch die Ethikkommission des Fachbereichs Medizin der Goethe-Universität Frankfurt (Beschlussnummer E 205/19, Geschäftsnummer 19-327). Ziel der Studie war es, die Effizienz des Lehransatzes zu überprüfen. Dazu wurde die Bewertung der durchgeführten Fälle am Simulator mit der Lernzeit im EyesiNet korreliert, um zu prüfen, ob es eine Abhängigkeit der Lernerfolgskurve bei der Befundung von Krankheitsbildern von der Beschäftigungsdauer in EyesiNet gibt.

Die Teilnahme an der Studie erfolgte freiwillig. Eingeschlossen wurden Studierende im 10. Semester, welche bereits die Vorlesungen der Augenheilkunde besucht hatten und das Augenheilkundepraktikum im Rahmen ihres klinischen Studienabschnittes durchliefen. Es wurde bei allen Studienteilnehmern vor Einschluss in die Studie deren Einverständniserklärung eingeholt. Die Studienteilnehmer wurden darüber aufgeklärt, dass ihre Zeit im EyesiNet pseudonymisiert gemessen und mit den Ergebnissen an den Simulatoren korreliert wird.

**Figure 3:**
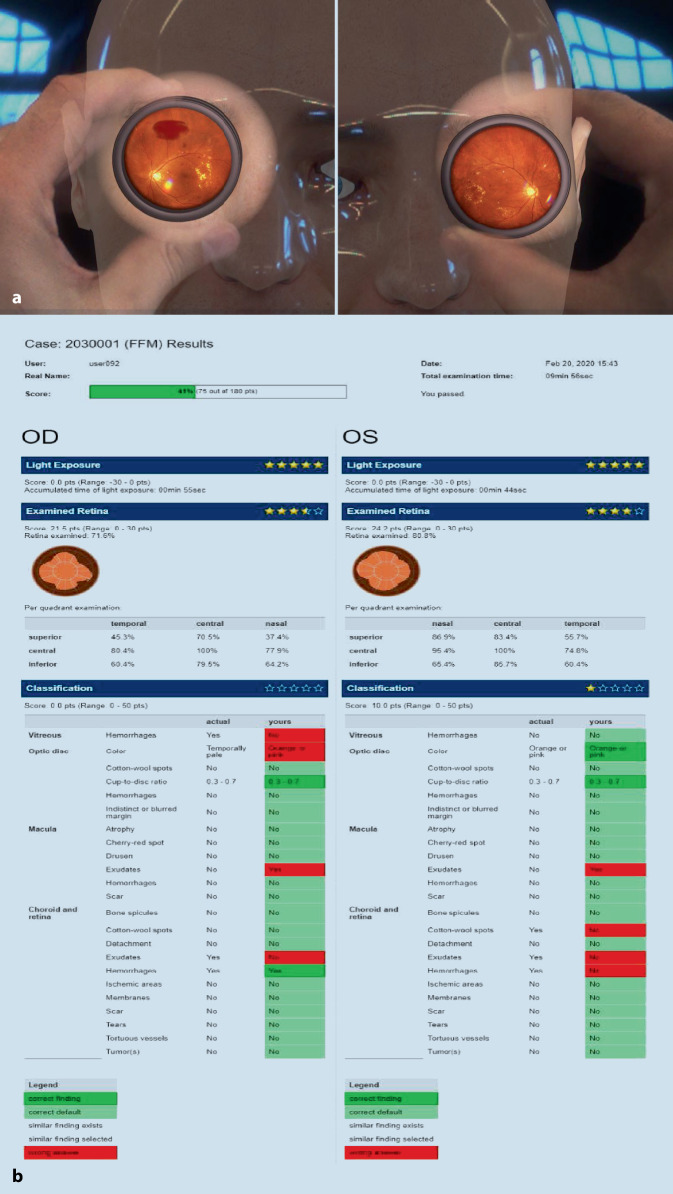

Am ersten Praktikumstag hörten die Studierenden zunächst einen 10minütigen Einführungsvortrag über die grundsätzliche Technik der Untersuchungen und bekamen eine kurze Demonstration der Simulatoren. Über eine Gesamtzugangszeit von 2 h konnten sie anschließend Fälle am Simulator untersuchen. Mithilfe ihres individuellen Zugangscodes konnten sie sich auf der Online-Plattform EyesiNet auf freiwilliger Basis begleitend weiter mit den dort aufgezeigten Pathologien beschäftigen. Am letzten Praktikumstag wurden am Simulator (Zugangszeit erneut 2 h) die erlernten Kenntnisse überprüft und die praktischen Fähigkeiten weiter vertieft. Bei jeder Einheit wurden den Studierenden dabei zufällig ausgewählte Fälle vorgestellt. Am Ende jedes Falles wurde am Simulator ein „Quiz“ mit Multiple-Choice-Fragen, Befundung und Diagnose bearbeitet, das sich auf in EyesiNet behandelte Inhalte bezog. Nach Bearbeitung des jeweiligen Falles wurde den Studierenden ihr Befundungsergebnis aufgezeigt (Abb. 3a, b), und sie hatten die Möglichkeit, den virtuellen Patienten nochmals zu spiegeln, um sich die Blickdiagnosen besser einprägen zu können. Mittels eines Fragebogens wurden EyesiNet sowie das Simulatortraining von den Studierenden nach Absolvierung ihres Praktikums bewertet.

## Erfassung der Punkte in Eyesi Direct und Indirect

Die Gesamtbewertung basiert auf folgenden Kriterien, die während der Untersuchung berechnet werden:

Lichtbelastung („light exposure“), Gesamtzeit der Untersuchung („examination time“), Fläche der untersuchten Netzhaut („examined retina“), Befunde („classification“) und Diagnose („diagnosis“) in Form von Multiple-Choice-Fragen [6].

Lichtbelastung:

Hier wird die Dauer von zu starkem und damit schädigendem Licht auf der Retina gemessen.

Gesamtzeit der Untersuchung:

Dies ist die Zeit, die ein Student für die Untersuchung benötigt. Bei der Untersuchungsdauer werden die Minuten gezählt.

Fläche der untersuchten Netzhaut:

Hier wird die untersuchte Fläche der Retina berechnet. Dabei wird die Retina als Fläche mit dem Wert 100 % angenommen und die relative Ausleuchtung berechnet.

Multiple-Choice-Fragen (Befunde und Diagnosen):

Hier wird zuerst anhand der richtigen und falschen Angaben der aktuelle Wert ermittelt, der dann zur Berechnung der Punktzahl dient:

$$\textit{Aktueller}\,Wert=\frac{\textit{Anzahl}\,\textit{richtige}\,\textit{Antworten}-\textit{Anzahl}\,\textit{falsche}\,\textit{Antworten}}{\textit{vorgegebene}\,\textit{Gesamtanzahl}\,\textit{richtiger}\,\textit{Antworten}}$$

Für jedes Bewertungskriterium sind Wertebereiche und Punktzahlen definiert, um den Messwert in eine Punktzahl zu transformieren. Dies wird gemäß der folgenden Formel linear interpoliert:

$$\textit{Relativer}\,Wert=\frac{\textit{Aktueller}\,Wert-\textit{Startwert}}{\textit{Endwert}-\textit{Startwert}}$$

$$\textit{Punktzahl}=\textit{Startpunktzahl}+\textit{Relativer}\,\textit{Wert*}\left(\textit{Endpunktzahl}-\textit{Startpunktzahl}\,\right)$$

Falls der aktuelle Wert außerhalb des Wertebereichs liegt, wird stattdessen die Minimal- bzw. Maximalpunktzahl angenommen. Da keine negativen Punkte vergeben werden, erhalten die Studierenden am Ende eine Gesamtpunktzahl von mindestens 0 Punkten und höchstens 100 Punkten (Abb. 3).

Die Werte- und Punktebereichte für die einzelnen Bewertungskriterien sind in Tab. 1 dargestellt und mit einem Musterprobanden versehen.

**Table 1:** 

Bewertungskriterium	Start- und Endwert	Start- und Endpunktzahl	Beispiel	
			Akt. Wert	Punktzahl
Untersuchungszeit	5–10 min	0 bis −10 pts	12 min	−10 pts
Lichtbelastung	2–10 min	0 bis −30 pts	1 min	0 pts
Untersuchte Netzhaut	0–100 %	0 bis 30 pts	80 %	24 pts
Befunde	0–100 %	0 bis 50 pts	50 %	25 pts
Diagnosen	0–100 %	0 bis 20 pts	20 %	4 pts
Gesamt/Ergebnis	–	0 bis 100 pts	–	43 pts

## Statistik

In dieser nicht randomisierten prospektiven Studie wurde gemäß den Vorgaben der Ethikkommission keine Kontrollgruppe gebildet, sodass alle Studierenden gleichermaßen die Möglichkeit hatten, sich in EyesiNet weiterzubilden. Ob und wie lange die Studierenden die Plattform nutzten, wurde ihnen auf freiwilliger Basis selbst überlassen. So konnte bei Abschluss der Studie eine nicht randomisierte Kontrollgruppe gebildet werden mit denjenigen Studierenden, die die Plattform nicht nutzten (Gruppe OHNE Training).

Die Daten wurden mithilfe der individuellen Zugänge an den Eyesi-Simulatoren erfasst und in Microsoft Excel 2016 sowie in BiAS Version 11.12 für Windows (epsilon-Verlag, Dr. rer. med. Hanns Ackermann, Goethe-Universität Frankfurt, Deutschland) ausgewertet.

Zum Vergleich, ob eine signifikante Verbesserung durch die EyesiNet-gestützte Weiterbildung erreicht werden konnte, wurde bei Vorlage einer nicht parametrischen Datenlage ein Wilcoxon-matched-pairs-Test für beide Gruppen angewendet sowie deren Effektstärke nach Rosenthal [7] bewertet. Die Abhängigkeit der Verbesserung von der in EyesiNet verbrachten Zeit wurde mittels Spearman-Rangkorrelation überprüft. Zum Vergleich der Vor-Werte beider Gruppen wurde ein Wilcoxon-Mann-Whitney-U-Test angewendet, um zeigen zu können, dass beide Gruppen die gleichen Startvoraussetzungen nach Besuch der Vorlesung und der Klausur hatten.

## Statistische Auswertung

Es konnten insgesamt 86 Studierende ausgewertet werden, wovon 32 Studierende das freiwillige Angebot nutzten und in ihrer freien Zeit während des Praktikums an der Online-Plattform EyesiNet trainierten (Gruppe MIT Training). Die aufgezeichnete Aktivität ergab, dass im Durchschnitt 28-mal (min. 1, max. 85) die Übersichtsseiten der 14 Pathologien aufgerufen wurde. Die Unterseiten (mit Detailinformationen) wurden im Durchschnitt von den Trainierenden 14-mal (min. 0, max. 42) aufgerufen.

Aus den Nicht-Trainierenden (*n* = 54) konnte bei der Auswertung eine nicht randomisierte Kontrollgruppe gebildet werden (Gruppe OHNE Training). Ein Loss-to-Follow-up trat bei 14 Studierenden auf.

## Ergebnisse

### Ergebnisse Eyesi Direct

Prüft man die Startvoraussetzungen der beiden Gruppen (OHNE vs. MIT Training) mittels Wilcoxon-Mann-Whitney-U-Test, so zeigt sich, dass beide Gruppen keinen signifikanten Unterschied aufwiesen (*p* = 0,29). Dies bedeutet, dass keine der beiden Gruppen vor dem Training mit EyesiNet einen Wissensvorsprung hatte.

Von den *n* = 54 Studierenden, welche nicht am EyesiNet-Training teilnahmen, wurden am Beginn des Praktikums 141 Fälle und am Ende des Praktikums insgesamt *n* = 138 Fälle am Eyesi Direct bearbeitet. Dabei lag bei einer Gesamtpunktzahl von 100 Punkten vor dem Training der Median bei 37 Punkten, danach konnte eine Steigerung auf einen Median von 44 Punkten erreicht werden. Beim Test der Nullhypothese konnte mit *p* = 0,02 im Wilcoxon-Matched-Pairs-Test eine signifikante Verbesserung mit einer Effektgröße von 0,1 festgestellt werden. Dies entspricht nach Rosenthal einem geringen Effekt.

Von den *n* = 32 Studierenden, welche am EyesiNet-Training teilnahmen, wurden am Beginn des Praktikums 93 Fälle und am Ende des Praktikums *n* = 83 Fälle am Eyesi Direct bearbeitet. Dabei lag bei einer Gesamtpunktzahl von 100 Punkten vor dem Training der Median bei 35 Punkten, danach konnte eine Steigerung auf einen Median von 45 Punkten aufgezeigt werden. Beim Test der Nullhypothese konnte mit *p* = 0,0004 im Wilcoxon-Matched-Pairs-Test eine hoch signifikante Verbesserung mit einer nach Rosenthal mittleren Effektgröße von 0,3 festgestellt werden.

Die Zeit des Trainings am EyesiNet korreliert dabei nach der Spearman-Rang-Korrelation mit *p* = 0,05 (Korrelationskoeffizient rho= 0,36) mit der Verbesserung am Eyesi Direct (Gesamtpunktzahl nachher – Gesamtpunktzahl vorher).

### Ergebnisse Eyesi Indirect

Im Wilcoxon-Mann-Whitney-U-Test zeigt sich, dass beide Gruppen zu Beginn des Praktikums wie im Eyesi Direct keinen signifikanten Unterschied in den Ergebnissen am Eyesi Indirect aufwiesen (*p* = 0,10).

Von den *n* = 54 Nicht-Trainierenden wurden am Beginn des Praktikums 147 und am Ende des Praktikums 133 Fälle am Eyesi Indirect bearbeitet. Dabei lag bei einer Gesamtpunktzahl von 100 Punkten vor dem Training der Median bei 22 Punkten, danach konnte eine minimale Steigerung auf einen Median von 23 Punkten erreicht werden. Dies entspricht mit *p* = 0,41 im Wilcoxon-Matched-Pairs-Test keiner signifikanten Verbesserung im Verlauf.

Von den *n* = 32 Trainierenden wurden am Beginn des Praktikums 87 Fälle und am Ende des Praktikums *n* = 85 Fälle am Eyesi Indirect bearbeitet. Dabei lag bei einer Gesamtpunktzahl von 100 Punkten vor dem Training der Median bei 25 Punkten, danach konnte eine Steigerung auf einen Median von 26 Punkten erreicht werden. Damit konnte mit *p* = 0,17 im Wilcoxon-Matched-Pairs-Test keine signifikante Verbesserung aufgezeigt werden.

Nach der Spearman-Rang-Korrelation korreliert die Zeit des EyesiNet-Trainings mit *p* = 0,12 knapp nicht mit der Verbesserung am Eyesi Indirect (Gesamtpunktzahl nachher – Gesamtpunktzahl vorher).

## Analyse der einzelnen Protokollwerte

Die Auswertung der einzelnen Bewertungskriterien, welche zur Gesamtbewertung am Eyesi Direct und Indirect führen, ist in Tab. 2 und 3 dargestellt.

**Table 2:** 

Eyesi Direct	Gruppe MIT Training		Gruppe OHNE Training	
	Tag 1 MW±SD	Tag 4 MW±SD	Tag 1 MW±SD	Tag 4 MW±SD
*Gesamtpunktzahl*	*41,02* *±* *16,08*	*50,31* *±* *22,07*	*42,96* *±* *18,83*	*47,91* *±* *20,54*
Lichtbelastung RA	−0,91 ± 3,30	−0,95 ± 2,36	−0,50 ± 1,37	−0,15 ± 0,88
Lichtbelastung LA	−0,47 ± 1,53	−0,39 ± 1,29	−0,11 ± 2,64	−0,51 ± 3,57
Untersuchte Netzhaut RA	28,03 ± 4,76	28,50 ± 4,28	26,63 ± 6,21	27,31 ± 6,29
Untersuchte Netzhaut LA	26,53 ± 6,31	27,76 ± 5,24	25,94 ± 8,91	25,78 ± 7,71
Befunde RA	7,74 ± 12,72	11,81 ± 17,08	10,39 ± 13,80	13,12 ± 17,10
Befunde LA	9,26 ± 16,04	16,82 ± 21,12	11,54 ± 18,98	13,75 ± 19,96
Diagnose RA/LA	5,18 ± 6,70	7,36 ± 8,62	5,88 ± 7,91	6,51 ± 8,31
Gesamtzeit der Untersuchung	0,0 ± 0,0	0,0 ± 0,0	0,0 ± 0,0	0,0 ± 0,0

Microsoft Excel 2016

**Table 3:** 

Eyesi Indirect	Gruppe MIT Training		Gruppe OHNE Training	
	Tag 1 MW±SD	Tag 4 MW±SD	Tag 1 MW±SD	Tag 4 MW±SD
*Gesamtpunktzahl*	*27,70* *±* *13,71*	*32,64* *±* *19,87*	*24,29* *±* *14,74*	*26,27* *±* *15,88*
Lichtbelastung RA	−0,15 ± 0,83	−0,19 ± 0,84	−0,10 ± 1,90	−0,10 ± 0,49
Lichtbelastung LA	−0,06 ± 0,33	−0,10 ± 0,68	−0,08 ± 0,37	−0,03 ± 0,22
Untersuchte Netzhaut RA	16,28 ± 5,53	16,88 ± 5,21	13,32 ± 0,09	13,97 ± 7,36
Untersuchte Netzhaut LA	14,81 ± 6,16	15,32 ± 5,62	12,51 ± 5,63	13,08 ± 6,32
Befunde RA	6,30 ± 12,38	10,96 ± 17,53	7,01 ± 14,34	5,92 ± 13,97
Befunde LA	7,69 ± 13,22	7,92 ± 14,99	6,16 ± 12,61	8,22 ± 14,00
Diagnose RA/LA	6,02 ± 6,80	10,24 ± 8,45	6,47 ± 7,50	7,52 ± 7,74
Gesamtzeit der Untersuchung	0,0 ± 0,0	0,0 ± 0,0	0,0 ± 0,0	0,0 ± 0,0

Microsoft Excel 2016

Hier befinden sich die Studierenden immer in der erlaubten Gesamtzeit. Von Beginn an wird sowohl am Eyesi Direct als auch am Eyesi Indirect mit einer geringen Lichtbelastung für die Netzhaut gearbeitet. Während die Ergebnisse der Fläche der untersuchten Retina am Eyesi Direct im oberen Bereich liegen, liegen die Ergebnisse am Eyesi Indirect deutlich darunter. Eine Steigerung der Punktzahl kann v. a. im Bereich der Befundung und Diagnosestellung aufgezeigt werden.

## Auswertung der Fragebögen

**Figure 4:**
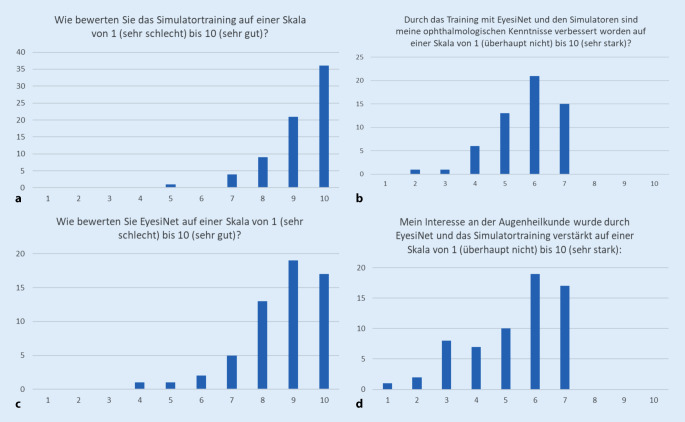

Nach Auswertung der Fragebögen zeigt sich, dass die Mehrheit der Studierenden sowohl das Training am Simulator als auch an EyesiNet überzeugt hat (Abb. 4) und sie auch subjektiv das Gefühl hatten, dadurch ihre ophthalmologischen Kenntnisse verbessern zu können. Zudem zeigte sich, dass das Interesse an dem im Studium eher kleinen Fachgebiet „Ophthalmologie“ durch ein an die Bedürfnisse der Studierenden angepasstes Training weiter gesteigert werden kann.

## Diskussion

Es konnte gezeigt werden, dass die Kombination aus praktischem Training an Simulatoren und begleitender, in das praktische Training auf geeignete Weise verwobener Theorie im Rahmen von Online-Plattformen nicht nur subjektiv das Interesse und die ophthalmologischen Kenntnisse der Studierenden steigert, sondern auch nachweislich zu besseren Ergebnissen in der Ausbildung führt.

Gerade die praktischen Fertigkeiten dürfen im Rahmen eines Medizinstudiums nicht zu stark in den Hintergrund rücken. Oft fehlt den Assistenzärzten dann in der Praxis die Fähigkeit, ihr theoretisches Wissen anzuwenden, die erforderlichen diagnostischen Tätigkeiten selbst umzusetzen und nach Erstellung der richtigen Diagnose die zutreffenden Behandlungsmethoden einzuleiten.

Anhand der Simulation erhalten die Studierenden am ersten Praktikumstag ein objektives Bild über ihren aktuellen Wissensstand in der Ophthalmologie und durch das Erlernen der Fertigkeiten auch die Motivation, dadurch bei genügend theoretischem Wissen auch selbstständig die richtige Diagnose stellen zu können.

Wie sich anhand der oben genannten Ergebnisse zeigt, ist gerade die direkte Ophthalmoskopie eine relativ einfache Untersuchungsmethode, die verstärkt im Unterricht der Studierenden eingesetzt werden sollte.

Hier ist bereits ein Lerneffekt allein durch die Praxis am Simulator für die direkte Ophthalmoskopie nachweisbar. Deutlich höher fällt er jedoch aus, wenn die Studierenden in der Zwischenzeit die Befunde an der Online-Plattform aufgearbeitet haben.

Das Erlernen dieser Fertigkeiten zeigt sich als wesentlich einfacher als das Erlernen der indirekten Ophthalmoskopie und sollte gefördert werden, da die direkte Ophthalmoskopie in der späteren beruflichen Laufbahn auch von Internisten, Pädiatern und Hausärzten einfach durchgeführt werden kann. Sie kann dabei helfen, Notfälle zu filtrieren (wie u. a. Zentralarterienverschluss, Stauungspapille …) und den richtigen Fachdisziplinen zuzuordnen [8].

Bei der indirekten Ophthalmoskopie konnte keine signifikante Verbesserung während des Praktikums erreicht werden: Die Ergebnisse am Eyesi Indirect sind sowohl beim primären als auch sekundären Simulationstraining bei beiden Gruppen deutlich schlechter als die Trainingsergebnisse am Eyesi Direct, was die Vermutung nahelegt, dass bei der indirekten Ophthalmoskopie eine bedeutend höhere Lernzeit bei der Anwendung benötigt wird, um Strukturen darzustellen und dann auch richtig bewerten zu können. Dies liegt mutmaßlich daran, dass die Studierenden erst ein Gefühl für die Augen-Hand-Koordination sowie für das invertierte Bild entwickeln müssen. Die hier vorhandene Trainingszeit von insgesamt 4 h kann aber den Studierenden zumindest helfen, ein Gefühl für diese Methodik zu entwickeln [9] und ggf. weiteres Interesse an der Augenheilkunde wecken und damit auch zur Entscheidungsfindung für die individuell richtige Fachdisziplin beitragen.

Die Kombination, dies mit jederzeit zugänglichen Online-Inhalten zu verknüpfen (welche z. B. auch über Mobiltelefon und Tablet zugänglich sind), ergibt die Möglichkeit, dass die Studierenden auch nach dem Praktikum immer wieder auf diese Informationen zurückgreifen und die erarbeiteten Lerninhalte als eine Art Nachschlagewerk nutzen können.

Dies kann z. B. dann helfen, wenn Studierende ein Erkrankungsbild zwar als bekannt erkennen, aber die Zuordnung zur Pathologie nicht mehr herstellen können.

Dass diese Funktion genutzt wird, konnte in dieser Studie aufgezeigt werden. So wurde das Portal auch nach dem abgeschlossenen Praktikum von 10 der 32 Studierenden in der Trainierenden-Gruppe weiter aufgerufen. Dagegen wurde das Portal nur von einer studierenden Person aus der Gruppe derjenigen genutzt, die sich in der Praktikumszeit nicht mit EyesiNet beschäftigt hatten. Eine Schwäche dieser Studie ist die begrenzte Zeit, die den Studierenden in 1 Woche zur Erlernung dieser Fähigkeiten zur Verfügung steht. Dementsprechend ist es überraschend, dass in der direkten Ophthalmoskopie trotzdem eine signifikante Verbesserung der Ergebnisse erreicht werden konnte. Es konnte jedoch nur eine studierende Person nach dem Training am Eyesi Direct die Maximalpunktzahl von 100 Punkten in einem Fall erreichen. Die Maximalpunktzahl am Eyesi Indirect waren 88 Punkte. Ausbildungsziel sollte sein, einen Großteil der Studierenden auf dieses hohe Niveau bringen zu können. Hierbei ist der Lernaufwand eines jeden Studierenden individuell einzustufen. Gerade aber hierfür sind Simulatoren vorteilhaft, da sie den Studierenden ermöglichen, nach Beendigung ihres Praktikums eigenständig weiter zu trainieren. Hierfür bieten wir den Studierenden Trainingszeiten an den Simulatoren an – und natürlich auch die Möglichkeit, im Wahlfach Ophthalmologie weiter ihre Kenntnisse zu vertiefen.

Mit *n* = 14 kam es zu einer relativ hohen Loss-to-Follow-up-Rate. Diese Rate basiert unter anderem auf dem sehr umfangreich curricular verankerten Ausbildungsprogramm. So kam es zu Überschneidungen mit anderen Fächern, was dazu führte, dass einige der Studierenden ihren Fehltag am letzten Praktikumstag nahmen. Dies kann neben mangelndem Interesse auch der Grund dafür sein, dass es nicht zu einer idealen Teilnahme der Studierenden an dem freiwilligen Angebot der Online-Courseware EyesiNet kam. Von den 86 Studierenden machten nur 32 Studierende davon aktiven Gebrauch. Es ist zu überlegen, ob das bisher freiwillige Online-Training in einen Pflichtteil während des Praktikums umstrukturiert werden sollte, sodass alle Studierenden auf den gleichen Stand gebracht werden können. Des Weiteren muss stärker auf eine sinnvolle Anordnung der Praktika und Prüfungen geachtet werden. Hierbei werden wir auch dem Wunsch der Studierenden nachkommen und in Zukunft die Online-Plattform bereits während der Vorlesungszeiten zur Verfügung stellen, sodass diese sowohl zur Klausur- als auch zur Praktikumsvorbereitung genutzt werden kann.

## Schlussfolgerung/„Fazit für die Praxis“

- Die Kombination aus realistischer Simulation und den dazu passenden Lerninhalten auf Online-Plattformen ist motivierend und effizient. Sie führt sowohl subjektiv als auch objektiv zu verbesserten ophthalmologischen Kenntnissen.
- Es ergibt sich daraus die Möglichkeit, die Ausbildung sowohl vor, während als auch nach der Absolvierung des Praktikums fortzuführen, da der Zugang den Studierenden weiterhin zur Verfügung steht und das „Modul“ sowohl vor dem abschließenden Examen mit in die Vorbereitungen integriert werden kann oder als mobile App im Berufsleben jederzeit und an jedem Ort wieder aufrufbar ist.
- An der Augenklinik der Goethe Universität Frankfurt am Main ist die Kombination aus dem Angebot des Online-Trainings in Kombination mit der Simulationsausbildung ein fester Bestandteil des Praktikums geworden und soll in einem weiteren Lehrantrag auf die Befundung am Vorderabschnitt ausgeweitet werden.
- In Zeiten der COVID-19-Pandemie kann durch Simulatortraining eine sichere Umgebung für praktische Übungen geschaffen werden. Überdies können mit EyesiNet auf digitale Weise interaktive Zusatzinformationen zu den Lehrbüchern zur Verfügung gestellt werden. Bei Bedarf bieten wir an, EyesiNet auch anderen Weiterbildungshäusern zur Verfügung zu stellen.
